# Supplementary material for: Fuzzy-Modulus-Based Layered Elastic Analysis of Asphalt Pavements for Enhanced Fatigue Life Prediction
Source: Materials (Basel). 2025 Jun 26;18(13):3034. doi: 10.3390/ma18133034 (PMC12250932; doi:10.3390/ma18133034)
Supplement: Supplementary file 1 [file materials-18-03034-s001.zip › materials-3688791-supplementary.pdf]

File S1: Pseudocode for Fuzzy-Modulus-Based Pavement Fatigue Analysis

```
%% Define pavement parameters based on Asphalt Institute recommendations
Set air_void_content Va, void_content Vv
Calculate correction factor C using Va and Vv
Define fatigue life function Nf = f(epsH, EY)
Define rutting life function Nr = f(epsV)

%% Define fuzzy elastic moduli for asphalt layers (5-point fuzzy numbers)
For each asphalt layer (Wearing, Binder, Base):
   Set EY_min0, EY_min05, EY_mode, EY_max05, EY_max0

%% Define alpha-cut levels for fuzzy analysis
Set NG as number of alpha-cuts (e.g. NG = 51)
Generate alpha_levels as linearly spaced from 0 to 1

%% Fuzzy interval analysis loop
For each alpha in alpha_levels:
   For each asphalt layer:
      If alpha <= 0.5:
         EY_low = linear interpolation between EY_min0 and EY_min05
         EY_high = linear interpolation between EY_max0 and EY_max05
      Else:
         EY_low = linear interpolation between EY_min05 and EY_mode
         EY_high = linear interpolation between EY_max05 and EY_mode

  %% Pavement response calculations at interval boundaries
   For each bound (low, high):
      [epsH, epsV] = PavementResponseCalculation(EY_low or EY_high)
      Nz = fatigue life function evaluated at (epsH, EY)
      Nd = rutting life function evaluated at epsV

  %% Store min/max strain and life results for current alpha
   epsH_alpha_lower(alpha) = min(epsH)
```

```
    epsH_alpha_upper(alpha) = max(epsH)
    epsV_alpha_lower(alpha) = min(epsV)
    epsV_alpha_upper(alpha) = max(epsV)
    Nf_alpha_lower(alpha) = min(Nf)
    Nf_alpha_upper(alpha) = max(Nf)
    Nr_alpha_lower(alpha) = min(Nr)
    Nr_alpha_upper(alpha) = max(Nr)

%% Construct fuzzy envelopes for strains and life predictions
Create fuzzy number envelope for epsH, epsV, Nf, and Nr using alpha-level results

%% Defuzzification of fatigue and rutting life
For each life parameter (Nf, Nr):
    Apply defuzzification methods (centroid, bisector, MOM, SOM, LOM)
    Return scalar values for practical decision-making

%% Visualization of fuzzy sets and defuzzification results (optional)
Plot fuzzy envelopes and defuzzified values for interpretation

%% SUBFUNCTIONS
Function PavementResponseCalculation(E_moduli):
    %% Define layered pavement structure properties
    Set layer thicknesses, Poisson ratios, loading parameters
    Convert elastic moduli E_moduli to layer properties
    %% Formulate governing equations (layered elastic theory)
    Setup system of equations describing stresses and strains
    %% Apply boundary and continuity conditions
    Solve resulting equations numerically to obtain displacements and strains
    %% Evaluate critical strains
    Compute horizontal tensile strain (epsH) at bottom of asphalt layers
    Compute vertical compressive strain (epsV) at top of subgrade
    Return epsH, epsV
End function
```
